# Determinants and causes of maternal mortality in Iran based on ICD-MM: a systematic review

**DOI:** 10.1186/s12978-019-0676-y

**Published:** 2019-02-08

**Authors:** Rostam Zalvand, Maryam Tajvar, Abolghasem Pourreza, Hadi Asheghi

**Affiliations:** Department of Health Management and Economics, School of Public Health, Tehran University of Medical Sciences, Tehran, Iran

**Keywords:** Maternal mortality, MMR, ICD-MM, Determinants of maternal mortality, Causes of maternal death, Iran, Systematic review

## Abstract

**Background:**

No systematic review has explored the causes of and factors associated with maternal mortality in the context of Iran. This study reviewed determinants and causes of maternal mortalities during pregnancy, delivery and the puerperium using the International Classification of Diseases-Maternal Mortality (ICD-MM), introduced by the World Health Organization.

**Methods:**

A systematic electronic search of all the studies that identified causes and/or determinants of maternal deaths in any part of Iran or in the whole country were included, without any restriction of time or language of studies. To identify the studies to include in this study, a combination of hand searching and bibliographies was also conducted. These sources and citations yielded a total of 653 articles; nevertheless, only 29 articles met the inclusion criteria, hence, required data were extracted, summarized, and grouped together from these papers and are reported in the tables.

**Results:**

Amongst the 29 studies published between 2003 and 2017 in Iran, 24 studies were cross-sectional. Overall, 4633 deaths were reviewed, and 2655 (58%) of the cases included the data on the causes of death generally. According to the ICD-MM, a total of 69.9, 20.6, and 5.2% of the mortalities were due to direct, indirect and unspecified causes respectively and 4.3% of the causes were not clear in several studies. The leading direct and indirect causes of death were identified as hemorrhage (30.7%) and hypertensive disorders (17.1%) and circulatory system diseases (8.1%) respectively. Several factors including gravidity, type of delivery, socio-economic status of mothers, locations of birth, death and maternity care venues were found in the original studies as the most important determinant of maternal mortalities in Iran.

**Conclusions:**

This study, provided an updated summary of evidences on the causes and determinants of maternal death in Iran, which is critically important for the development of interventions and reduction of the burden of maternal mortality and morbidities.

## Plain English summary

The reduction of maternal death is a key international development goal. To reduce maternal deaths, valid information should be produced in order to help in policy making and development of plans aiming in improvement of maternal health and reduce maternal death. We conducted a systematic review to determine the prevalence causes of maternal deaths in Iran and also to provide a summary of evidences on the determinants of maternal death among Iranian mothers. After conducting a systematic search, 29 articles were identified that investigated causes and/or determinants of maternal deaths in Iran. A review of these studies indicated that overall 70% of deaths had a direct cause with hemorrhage as the leading cause consisting 31% of all the maternal deaths in Iran and 21% had an indirect cause, of which circulatory system diseases was the most common (8% of all deaths). The cause of rest of deaths (10%) were either unspecified or unclassified causes. Several factors including gravidity, type of delivery, socio-economic status of mothers, locations of birth, death and maternity care venues were found as the most important determinants of maternal mortalities in Iran. Although, some progress in reduction of maternal death has already been achieved in Iran, especially in the past decade, further improvements are still needed to reduce deaths to the lowest possible rate.

## Background

Maternal death has important negative social and economic consequences on the society, and on the health and lives of family, especially on the new born babies, particularly under conditions of socioeconomic deprivation [1, 2]. The Maternal Mortality Ratio (MMR; number of maternal deaths per 100,000 livebirths), is an important public health indicator that reflects both the quality of health care services and the women’s status and importance in their society [3, 4]. The priority accorded to reductions in maternal mortality is shown by its choice as one of the eight Millennium Development Goals (MDGs). MDG5 focused on improving maternal health with two clear sub-goals, first, a 75% reduction in the maternal mortality ratio between 1990 and 2015 and second, to obtain universal access to reproductive health for women. Political attention to how countries are progressing towards MDG5 targets is intensifying [5–7]. However, despite a global trend of declining maternal mortality, most of the countries have been behind to achieve their MDG targets by 2015 [3]. A comprehensive assessment of global trends in maternal mortality in 2010 suggested that the MMR had decreased by 1.3% per year since 1990, which is lower than the 5.5% recommended by MDG5 [8]. There is evidence that, despite the considerable decline in MMR in the world, it has arisen from the risks attributable to pregnancy and childbirth as well as from the poor quality care of health services, which needs serious attention [9].

Iran is one of the successful countries in achieving MDG5 with 75% reduction in MMR by the year 2015, reaching to the highest reduction compared to neighboring countries except Turkey. In 1975, Iran’s MMR was 274, fallen to 150 in 1990 and a continuous decline to 94 in 1995, 38 in 2005, 30 in 2008 and 25 in 2015, a figure comparable with developed nations [3, 10, 11]. Nevertheless, despite this achievement, the current MMR in Iran, its main causes and determinant factors are still worthy of consideration, particularly as there is evidence that the direct causes are still the major reasons for maternal death in Iran [12], and similarly Iran is experiencing a paradigm shift in its major causes of maternal mortality from those more commonly seen in developing nations than those in developed nations [3].

In this study, we aimed to systematically review, first, the causes of maternal death in Iran and second, the determinants of maternal mortality in the country. The preliminary result of our review indicated that no systematic review has been previously conducted on the determinants or causes of maternal mortality in Iran. The existing literature reports inconsistent results and most of them focused on special areas in Iran [1], particular groups of women or a limited period [12]. The only study that systematically reviewed the causes of maternal mortality in Iran is the recent study of Dadipoor and colleagues [13], which focused on 19 original studies divided into direct and indirect causes, and without using any framework classified the causes of death. To report the cause of maternal death in Iran, we used the framework of the ICD-MM, developed by the World Health Organization (WHO), to prevent under-reporting and specify the correct code to the causes of maternal death and improve the quality of the data. As a result of the lack of such framework, inadvertent errors such as misclassification and misinterpretation of the coding rules of the cause of death, data on the causes of death can be especially difficult to analyze. ICD-MM helps to improve comparability of the data, making it possible to link multiple data sources and create an integrated information system for countries, which is the basis of the interventions to reduce the incidence of mortalities [14]. The ICD-MM will standardize documentation and analysis related to maternal, direct and indirect causes of death and their attributes [15].

Unfortunately, there is insufficient current updated comprehensive evidence on the determinants of maternal death in the context of Iran and the magnitude of its various causes. Nevertheless, documentation of information on the MMR can help identify areas of socioeconomic inequity and serve as a barometer of any society’s health system [1, 16]. This review study aimed to provide an updated and inclusive evidence on the causes and determinants of maternal death in Iran, which is crucial for effective policy making, health programme decisions, funding to control the problem, underpin advocacy efforts, and to stimulate further research.

## Methods

### Criteria for considering studies for this review

All types of studies that aimed to identify either causes or determinants of maternal deaths or both in any part of Iran or in the whole country were included. For “maternal death”, we considered the definition of death by WHO [16] as “the death of a woman while pregnant or within 42 days of the termination of pregnancy; irrespective of the duration and site of the pregnancy, from any cause related to or aggravated by the pregnancy or its management but not from accidental or incidental causes”. No time or language limitation was considered in searching for studies.

### Search strategy

To identify studies based on the inclusion criteria of this review, firstly, an electronic literature search was conducted in “PubMed”, “Scopus”, “Web of Science”, “Cochrane library databases”, “Google” and “Google scholar”. In the PubMed and Cochrane library databases, we initially searched by Meshing “Maternal Mortality”, then “Iran” was added to the search terms as follows: *Mortality, Maternal [Title/Abstract] OR Maternal Mortalities [Title/Abstract] OR Mortalities, Maternal [Title/Abstract]) AND Iran [Title/Abstract]*. In other databases, all the synonyms of “maternal mortality” were searched and afterwards “Iran” was also added to the search terms. This was the most conservative way of searching in this review, because “maternal mortality” was a popular term and could cover any sub-heading, including causes or determinant of maternal mortality.

To ensure that all the relevant studies including those published in Farsi (formal language of Iran) was identified, an electronic search in Farsi was conducted in Google and “Google scholar” as well as in the main national databases including “SID”, “MagIran” and “IranMedex”, using the translation of “maternal mortality” and its various synonyms in Farsi and also “Iran” as keywords.

Additionally, the bibliographies of the included studies were also searched. We scanned the reference lists of all the included papers to identify the main contributing authors to this topic. A further search was then made using the names of these authors in order to ensure that the most relevant studies were included in the review. Similarly, hand-searching was conducted among very relevant journals, grey literature, government documentation, informal reports, these, etc. All the studies discovered through searching were entered in to EndNote × 7, for further investigation on their relevance to the topic.

### Study selection

**Fig. 1:**
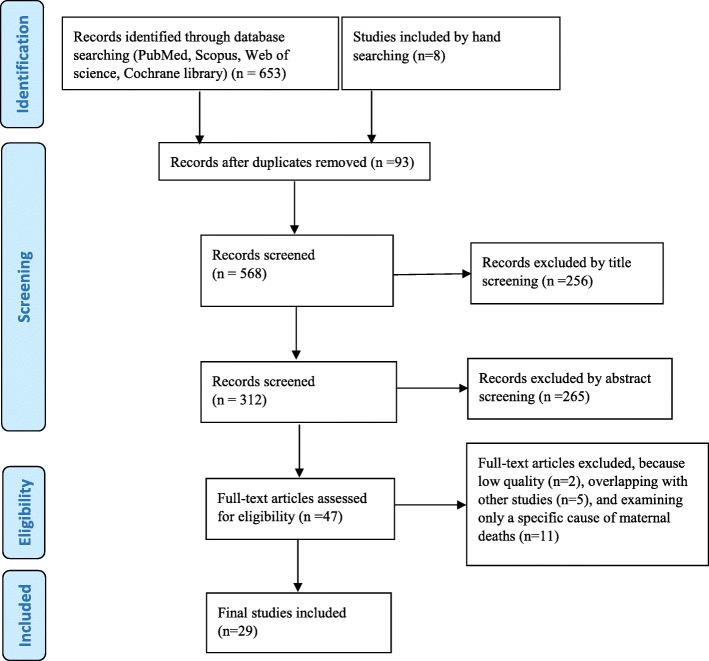
PRISMA flow diagram illustrating article selection and elimination

The process of article identification and selection of papers is shown in Fig. 1 below. Initially 653 papers found through electric and 8 papers found through hand searching. The papers that duplicated two or more times (*n* = 93) were eliminated and 568 papers remained for further selection. Then, screening of titles and abstracts was independently conducted by two reviewers and the studies not relevant to the research’s aim were excluded. Finally, 47 papers were remaining for full-text assessment to check for eligibility and out of these papers, 11 articles were excluded because they only investigated one specific cause of maternal deaths, for example burning in pregnancy [17]. Furthermore, five of the studies only shared datasets with previous studies, thus provided same results, therefore, they were also excluded [18–22]. The study of Dadipoor and colleagues [13], the only systematic review conducted in Iran, was also excluded because of its weaknesses mentioned earlier and the fact that it shared the original studies included in this review. Finally, 29 studies met the inclusion criteria for this review study, hence were utilized. A summary of the included studies are available in Table 1.

**Table 1:** Summary of characteristics and findings of the included studies (ordered chronologically)

Ref. Langu.	Sample, setting, time, design, analysis	Main Findings	Quality score (0–22)
Farzianpour et al. 2017 [35]. English	*N* = 109 Tehran Province 2011–2015 XS (descriptive)	Descriptive: Residence (rural 38%, urban 56%, unknown = 6%), Education (primary 20%, high school 30%, university 50%), Occupation (employed 71%, unemployed 29%), Main insurance (social security 50%, public health service 34%, private 16%), Supplemental insurance (yes 27%, no 73%), Economic status (high 25%, middle 10%, poor 65%), Time of death (pregnancy 25%, delivery 9%, post-delivery 66%), Place of death (home 2%, in transportation 5%, health facility 93%) Most frequent cause: Hypertension (23.6%)	18
Shahidi et al. 2017 [36]. Farsi	*N* = 20 Sabzevar city 2003–2013 XS (bivariate) χ2 test	Descriptive: Mean age 31, Mean gravity 2.6, Residence (Urban 70%, rural 30%), Type of delivery (ND 40%, CS = 60%) Most frequent cause: Hemorrhage (30%), Underling disease (30%) Determinants: Associated [Residence, *p* = 0.04]	18
Jamshidi et al. 2016 [37]. English	*N* = 70 Khuzestan Province 2009–2013 XS (descriptive)	Descriptive: Mean gravity 2.9, Mean parity 1.7, Residence (urban 54.3%, rural 45.7%), Occupation (employed 6%, unemployed 94%), Education (illiterate 13%, primary 66%, high school 13%, university 9%), Attended pregnancy (wanted 84%, unwanted 11%, unknown 5%) Most frequent cause: Hemorrhage (31.4%)	17
Karimzaei et al. 2016 [24]. English	*N* = 34 Iranshahr city2009–2013 XS (bivariate) χ2 test	Descriptive: Mean age 30, Mean gravity 3.9, Type of delivery (ND 50%, CS 18%, undelivered 32%), Residence (rural 65%, urban 35%), Education (illiterate 47%, primary 29%, high school 15%, university 9%), Place of birth (hospital 50%, home 9%, labor facility 9, undelivered 32%), Birth attendant (skilled 53%, trained 3%, traditional 6%, undelivered 32%), Economic status (high 38%, middle 29%, poor 32%) Most frequent cause: Hemorrhage (38.2%) Determinants: Associated [Gravidity, (*p* = 0.005)], NOT associated [Residence, type of birth, birth attendant, mother’s age and place of birth]	19
Vahiddastjerdy M et al. 2016 [12]. English	*N* = 896 Iran 2009–2012 XS (descriptive)	Descriptive: Mean age 30, Type of delivery (ND 26%, CS 44%, others 30%), Time of death (during pregnancy/childbirth 26%, postpartum 74%), Education (illiterate 18%, primary 72%, university 10%, no response 6%), Place of birth (hospital 67%, home 3%, on the way 2%, no response 28%), Economic status (high 1%, middle 3%, poor 90%, unknown 5%), Occupation (unemployed 91%, employee 9%) Most frequent cause: Hemorrhage (24.2%)	17
Kamiabi et al. 2015 [38]. Farsi	*N* = 1805 Iran 2007–2012 Ecological study (multivariate) Regression	Descriptive: Type of delivery (ND 29%, CS 61%), Education (illiterate 24%, literate 76%), Place of birth (health facility 93%, & home and way 7.2%), Birth attendant (skilled and trained 94%, traditional 6%), Prenatal care (yes 94%, No 6.3%), Completed prenatal care (Yes 66%, No 34%) Determinants: Associated [Completed prenatal care, Type of delivery, Education, Birth place, Birth attendant, Prenatal care, Improved water source], NOT associated [Male literacy, Use of improved sanitation, Attendance of girls in secondary schools]	19
Karimi-Zarchi et al. 2015 [39]. English	*N* = 40 Yazd province 2002–2011 XS (descriptive)	Descriptive: Mean age 29, Mean gravidity 2.6, Type of delivery (ND 20%, CS 55% & others 25%), Education (under diploma 40% & diploma or higher 60%), Place of death (hospital 92.5% & home 7.5%), Birth attendant (General practitioner 82.5%, trained midwife 12.5% & Local Midwife/Untrained Midwife 5%), Prenatal care (yes 77.5%, & No 22.5%) Most frequent cause: Hemorrhage (30%)	15
Tirkesh et al. 2015 [40]. Farsi	*N* = 22 Dezful 2007–2012 XS (bivariate) χ2 & ANOVA tests	Descriptive: Mean age 28, Period of death (during pregnancy 50% & postpartum 50%), Residence (rural/nomads 68% & urban 32%), Place of death (hospital 92% & home 18%) Most frequent cause: Underling disease (40.9%) Determinants: Associated [maternal age (*p* = 0.02)], NOT Associated [residence, type of delivery, place of maternal death and period of death]	16
Gholampour H. 2015 [41]. Farsi	Iran 2008–2013 Panel study χ2 test	Determinants: Associated [Fertility rate(p = 0.00), family income(*p* = 0.006), population(*p* = 0.004)], NOT Associated [Urbanization, hospital beds, number of midwifes & number of physician]	18
Rajai et al. 2014 [42]. Farsi	*N* = 91 Hormozgan province 2005–2012 XS (bivariate) χ2 & ANOVA	Descriptive: Mean age 30, Mean gravidity 3.8, Type of delivery (ND 43%, CS 37% & others 20%), Period of death (during pregnancy 23% & postpartum 77%), Residence (rural/nomads 60% & urban 40%) Most frequent cause: Hemorrhage (38.2%) Determinants: Associated [Type of deliveries (*p* = 0.001), risk factors during pregnancy (*p* = 0.022) & gravidity (*p* = 0.025)], NOT Associated [Residence, mother’s age and place of death]	17
Zokaei et al. 2014 [43]. Farsi	*N* = 79 Kurdistan province 2001–2013 XS (descriptive)	Descriptive: Mean age 35, Type of delivery (ND 37%, CS 52% & undelivered 11%), Period of death (during pregnancy 99% & unknown1%), Residence (rural/nomads 52% & urban 48%), Education (illiterate 66%, Under diploma29% & diploma and higher 5%), Place of birth (hospital 90%, home 8%, labor facility1% & in the way 1%), Pregnancy intentions (wanted 71%, unwanted19% & unknown10%) Occupation (housewife 98.7%, & employee 1.3%) Most frequent cause: Hemorrhage (34.2%)	17
Farrokh-Eslamlou et al.2014 [44]. English	*N* = 183 West Azerbaijan Province 2002–2011 XS (descriptive)	Descriptive: Type of delivery (ND 42%, CS 34% & others 24%), Period of death (during pregnancy 24% & postpartum 76%), Place of death (hospital 56%, in the way/home 20% & unknown24%), Pregnancy intentions (wanted 61% & unwanted39%), Prenatal care: (yes 80%, & No 20%) Most frequent cause: Hemorrhage (29.6%)	18
Poorolajal et al. 2014 [45]. English	*N* = 185 Hamadan Province 2006–2014 case-control study Regression	Descriptive: Mean age 30, Type of delivery (ND 32%, CS 49% & others 19%), Residence (rural/nomads 43% & urban 57%), Place of death (hospital 86%, home 5%, in the way 3% & unknown 5%), Birth attendant (Specialist 49%, midwife 30% & undelivered21%) Most frequent cause: Hemorrhage (37.1%) Determinants: Associated [Maternal age (*p* = 0.049), gravidity (p = 0.02) and preterm (< 37) of gestational age (p = 0.001) overweight (*p* = .01)], NOT Associated [Type of delivery and birth attendant, underweight]	18
Jamshid Pour et al. 2014 [46]. Farsi	*N* = 99 Kermanshah Province 2001–2013 XS (bivariate) χ2 test andregression	Descriptive: Mean age 31, Mean gravidity 3.4, Type of delivery (ND 42%, CS 45% & Others 12%), Period of death (during pregnancy 18%, childbirth 3% & postpartum 79%), Residence (rural/nomads 33% & urban 67%), Pregnancy intentions (wanted 36%, unwanted 30% & unknown 33%) Most frequent cause: Hemorrhage (23.2%) Determinants: Associated [period of death, & residence (*p* = 0.008)], NOT Associated [maternal age, pregnancy care, & mothers at highly risk]	17
Mobasheri et al. 2014 [47]. Farsi	*N* = 28 Chaharmahal Bakhtaran Province 2002–2012 XS (descriptive)	Descriptive: Mean age 31, Type of delivery (ND 32%, CS 46% & others 21%), Period of death (during pregnancy14% & postpartum 86%), Residence (rural/nomads 71% & urban 29%), Education (illiterate 50%, primary level21.4, diploma 14.3%, university level14.3%) Most frequent cause: Hemorrhage (32.1%)	17
Zarean et al. 2014 [27]. Farsi	*N* = 67 Esfahan province 2006–2012 XS (descriptive)	Descriptive: Period of death (during pregnancy 30% & postpartum 70%) Most frequent cause: Hemorrhage (22.4%)	15
Heidari et al. 2014 [48]. Farsi	*N* = 84 Kerman province 2006–2012 XS (bivariate) Poisson regression	Descriptive: Type of delivery (ND 45% & CS 55%), Period of death (during pregnancy 21% & postpartum 79%), Residence (rural/nomads 60% & urban 40%), Place of death (hospital 88% & in the way/home 12%), Place of birth (hospital 83% & in the way/home 17%), Birth attendant (General practitioner 64%, trained/untrained midwife 13% & Midwife 23%) Most frequent cause: Eclampsia (21.4%) Determinants: Associated [type of delivery (*p* = 0.01), midwife as a birth attendant (p = 0.006), trained and untrained midwifes (*p* < 0.001), & place of birth (p = 0.04)], NOT Associated [residence, maternal age, place of death, & period of death]	16
Sarani et al. 2014 [49]. Farsi	*N* = 57 Sistan and Baluchestan province 2002–2014 XS (descriptive)	Descriptive: Mean age 33.5, Mean gravidity 3.8, Type of delivery (ND 49% & CS 51%), Period of death (during pregnancy 26% & postpartum 74%), Residence (rural/nomads 96% & urban 4%), Education (illiterate 16% & literate 84%) Most frequent cause: Embolism (19.2%)	15
Mohammadinia et al. 2013 [50]. Farsi	*N* = 307 Sistan and Baluchestan province 2002–2010 XS (bivariate) χ2 & ANOVA	Descriptive: Mean age 28, Mean gravidity 4.1, Type of delivery (ND 61% & CS 39%), Period of death (during pregnancy 21% & postpartum 79%), Residence (rural/nomads 71% & urban 29%), Education (illiterate 62% & literate 38%), Place of death (hospital 59%, home 16%, labor facility 9% & in the way 16%), Birth attendant (Specialist/General Practitioner/Midwife 47%, trained midwife 10%, Local Midwife/Untrained Midwife 21% & undelivered21%) Most frequent cause: Hemorrhage (39.5%) Determinants: Associated [type of birth (*p* = 0.03), birth attendant (p = 0.005) and place of maternal death (*p* < 0.001)], NOT Associated [Residence]	18
Zolala F et al. .2012 [51]. English	Iran 2001–2008 Ecological study multivariate analysis	Determinants: Associated [male literacy (p < 0.001), unemployment (*p* = 0.04), & female literacy (*p* = 0.02)], NOT Associated [Proportion of midwives, & Urban residency]	17
Maharlouei et al. 2012 [52]. English	*N* = 101 Fars province 2003–2010 XS (descriptive)	Descriptive: Mean age 30, Type of delivery (ND 36%, CS 61% & others3%), Period of death (during pregnancy 24%, childbirth5% & postpartum 71%), Residence (rural/nomads 50% & urban 50%), Place of death (hospital 82%, home 6% & in the way 12%), Pregnancy intentions (wanted 76%, unwanted17% & unknown7%) Most frequent cause: Hemorrhage (35.6%)	17
Tajik P et al. 2011 [53]. English	Iran 2004–2006 Ecological study Regression	Determinants: Associated [Human development index (p < 0.001),&difference in illiteracy rate in women and men(*p* = 0.03)], NOT Associated [Gini coefficient]	20
Abdollahipour et al. 2011 [54]. Farsi	*N* = 25 Ilam Province 2001–2011 XS (bivariate) Fisher, T, χ2 tests, regression	Descriptive: Period of death (during pregnancy/childbirth 20% & postpartum 80%), Residence (rural/nomads 48% & urban 52%) Most frequent cause: Hemorrhage (36%) Determinants: Associated [education, underling disease (*p* = 0.001), high risk pregnancy, gravidity and quality of care (p = 0.001)]	17
GholamiTaramsari et al. 2008 [55]. Farsi	*N* = 66 Kohkiluyeh and Boyerahmad Province 1997–2007 XS (bivariate) χ2 test	Descriptive: Mean age 29, Type of delivery (ND 59%, CS32% & others 9%), Period of death (during pregnancy 9%, childbirth 26% & postpartum 65%), Residence (rural/nomads 77%, urban 23%), Education (illiterate 82% & literate 18%) Most frequent cause: Hemorrhage (41%) Determinants: Associated [education (*p* < 0.001), residence (*p* < 0.001), type of delivery (*p* < 0.001), place of maternal death (*p* < 0.001) and period of death (*p* < 0.001)], NOT Associated [gravidity]	17
Farrokh-Eslamlou et al. 2006 [56]. Farsi	*N* = 96 West Azerbaijan province 2001–2005 XS (bivariate) Regression	Descriptive: Mean age 31, Period of death (during pregnancy 25%, childbirth 49%, postpartum 17% & unknown 9%), Residence (rural/nomads 25%, urban 66% & unknown 9%), Education (illiterate 71%, literate 20% & unknown9%), Place of birth (hospital 49%, home/in the way 51%) Most frequent cause: Hemorrhage (46%) Determinants: Associated [education (*p* = 0.001), maternal age (*p* < 0.01), gravidity (*p* = 0.001), immune pregnancy ratio (*p* < 0.01)]	18
Mansouri et al. 2005 [28]. Farsi	*N* = 17 Khorasan province 1999–2005 XS (descriptive)	Descriptive: Mean age 31, Mean gravidity 3.1 Most frequent cause: Embolism (35.3%)	15
Galyan Tehrani et al. 2004 [57]. Farsi	*N* = 55 Kurdistan province 1998–2003 XS (bivariate) χ2 test	Descriptive: Type of delivery (ND 44%, CS 31% & others25%), Residence (rural/nomads 65% & urban 35%), Education (illiterate 44%, literate 40% & unknown16%), Place of birth (hospital 44%, home 29%, in the way 2% & undelivered25%), Birth attendant (General Practitioner 31%, trained midwife 4%, untrained midwife 25%, Midwife 15% & undelivered25%), Occupation (housewife 91.7%, & employee 8.3%), Prenatal care (yes 65.5%, & No 34.5%) Most frequent cause: Hypertension (31.8%) & abortion (31.8%) Determinants: Associated [type of delivery (*p* = 0.0006), gravidity (*p* = 0.003), birth attendant (*p* = 0.0000), & pregnancy care (*p* = 0.0000)], NOT Associated [maternal age, place of birth, type of job, and education]	17
Mehrzad-Sadagiani et al. 2003 [58]. English	N = 66 West Azerbaijan Province 1995–1998 XS (descriptive)	Descriptive: Type of delivery (ND 71%, CS 12% & others 17%), Residence (rural/nomads 12% & urban 88%), Education (illiterate 97% & literate 3%), Birth attendant (Specialist 3%, General Practitioner 3%, trained midwife 42%, Midwife 9% & undelivered 42%) Most frequent cause: Hemorrhage (54.6%)	15
Akhlagi et al. 2003 [59]. Farsi	*N* = 31 Mashhad Province 1991–2001 XS (descriptive)	Most frequent cause: Hemorrhage (58%)	15

*XS* Cross sectional, *ND* Normal delivery, *CS* Cesarean section

### Quality assessment

Quality of the included studies were assessed using STROBE checklist, which includes 22 items, thus possible ranges of scores was 0–22 [23]. The quality assessment process was conducted independently by two reviewers of this study and discrepancies between them, which occurred only in 3 manuscripts, were resolved by discussion. The descriptive or analytical studies had to be scored at least 15 out of 22 to be included in this review study. Descriptive studies compared to analytical studies generally gained lower scores as the items numbers 7, 11, 12, 13, 17, and 14 could not be assessed in descriptive studies, but only in analytical studies.

### Data extraction and analysis strategy

Data required including MMR, reported cause, factors associated with maternal death including individual or ecological factors, were extracted from the articles using a purposefully designed data extraction form. In addition, information on study methods such as design, setting, sample size and characteristics, sampling and analyses were also extracted from the papers (Table 1). The process of data extraction was conducted by two of this study’s reviewers independently and discrepancies were checked and resolved by discussion accordingly.

For analyzing the extracted data, given the variety of types of studies (descriptive or analytical, individual level or ecological studies), variety of measures and methods and the variety of study population (from different cities or provinces, rural or urban areas), a formal meta-analysis was neither possible nor appropriate.

To report causes of maternal deaths, all the reported causes in the studies included were classified and grouped into four causes, according to ICD-MM [16], as direct, indirect, unspecified and coincidental causes. The direct causes include pregnancies with abortive outcome; hypertensive disorders in pregnancy, childbirth, and the puerperium; obstetric hemorrhage; pregnancy-related infection; other obstetric complications; and unanticipated complications of management groups. While the indirect causes include non-obstetric complications group, the unspecified causes include death during pregnancy, childbirth and the puerperium where the underlying cause is unknown or was not determined. The coincidental causes, one the other hand, include death during pregnancy, childbirth and the puerperium due to external causes. Nevertheless, because the definition of maternal death used in this study was adopted from the WHO, coincidental causes were excluded from the causes of maternal death, while calculating and/or reporting the denominator in respect to prevalence of direct, indirect and other unclassifiable maternal types of deaths.

Moreover, as the causes of death was not clearly reported, it was impossible to make classifications based on the ICD-MM in several of the included studies. Therefore, we added another group as “unclassified”, for example, where “other” causes of maternal deaths were not classified into direct or indirect in some of the included studies [24]. Similarly, to classify certain causes that had different codes at different groups, researchers held a meeting with clinical staff to classify those causes precisely.

## Results

### Description of the included studies

As shown in Table 1, of the 29 included studies, 13 studies (45%) were descriptive cross-sectional while 11 studies (38%) were both descriptive and analytical cross-sectional studies, 4 studies (14%) were ecological research, and 1 study (3%) had a case-control design. Similarly, 18 out of the 29 papers (62%) were written in Farsi and the others were written in English. The publication date of the included studies ranged from 2003 to 2017 and, overall, 4633 studies on deaths in this period were reviewed, of which 2655 deaths (58% of total deaths) had reported the cause of death.

Moreover, out of the 29 studies, 5 studies were conducted at the country level, 21 studies at the provincial level and 3 studies at the city level. The quality scores of the studies, ranged from 15 to 20, shown in Table 1, in the ranges of 0–22. Low quality studies [25, 26] were excluded.

### Causes of maternal deaths in Iran

In this review study, 25 out of the 29 studies gathered data on the causes of death from various sources including the Maternal Mortality Surveillance System (MMSS) in Iran, working with a panel of experts to determine the causes of all the maternal deaths that happened in the country. 16 out of the 25 studies included in this review, obtained the data on the cause of death from the MMSS. The source of the 8 studies were the death certificate of the mothers, indicating the causes diagnosed by the medical doctors. The last study was sourced from the autopsy findings.

The summary of results on the causes of maternal mortality is reported in Table 2. In the study of Zarean and colleagues [27], which had different findings from clinical and autopsy reports for causes of maternal mortality, the finding of autopsy reports was considered. Similarly, in the study of Mansouri and colleagues [28], which had different diagnosis on the causes of maternal death by the medical doctors and forensic medicine, the diagnosis of the medical doctors was considered in our review. As shown in the Table 2, out of the total 2655 deaths, the direct and indirect causes were 69.9 and 20.6% of cases respectively. Additionally, of all the reviewed deaths, unspecified causes of maternal deaths were responsible for 5.2% (18 of the deaths). Finally, the causes of the 4.3% of deaths were not reported clearly in the studies of this review.

**Table 2:** Causes of maternal mortality in Iran based on the ICD-MM

	Classification of causes of maternal deaths	N	%
Type	Group name/number		
Maternal death: direct	1. Pregnancies with abortive outcome	62	2.3
Maternal death: direct	2. Hypertensive disorders in pregnancy, childbirth, and the puerperium	458	17.3
Maternal death: direct	3. Obstetric hemorrhage	816	30.7
Maternal death: direct	4. Pregnancy-related infection	211	7.9
Maternal death: direct	5. Other obstetric complications^a^	282	10.6
Maternal death: direct	6. Unanticipated complications of management	26	1.0
	Total Direct	1855	69.9
Maternal death: indirect	7. Non-obstetric complications		
	• Diseases of the nervous system	3	0.1
	• Diseases of the circulatory system	217	8.1
	• Diseases of the respiratory system	26	1.0
	• Diseases of the digestive system	16	0.6
	• Diseases of the musculoskeletal system and connective tissue	4	0.2
	• Diseases of the genitourinary system	16	0.6
	• Other indirect causes	266	10.0
	Total Indirect	548	20.6
Maternal death: unspecified	8. Unknown/undetermined	138	5.2
	Unclassified (not reported)^b^	114	4.3
	Total	2655	100.0

^a^In this category, 142 cases equivalent to 5.3% of the total causes of maternal deaths were due to embolism

^b^This category is the cases that were not clear whether they died from other direct causes or other indirect causes

Out of the direct causes responsible for 1855 deaths (69.9%) in Iran, ‘obstetric hemorrhage’, was the most common cause, consisting 30.7% of the total deaths reported, followed by ‘hypertensive disorders in pregnancy, childbirth, and the puerperium’ as the second most common cause (17%). ‘Other obstetric complications’ (10.6%), ‘pregnancy-related infection’ (7.9%), ‘abortion’ (2.3%), and ‘unanticipated complications of management’ (1%) were respectively less important direct causes of maternal mortality (Table 2). However, it is worth mentioning that half of the “other obstetric complications” (Group 5 of the ICD-MM) was attributed to embolism. Similarly, out of the indirect causes, responsible for 20.6% of all the deaths (*n* = 548), deaths due to ‘circulatory system diseases’, was the most important cause, leading to 217 deaths (8.1% of total deaths). ‘Other indirect causes’ (10% of all the causes of deaths), including diseases of the nervous system, respiratory system, digestive system, genitourinary system, musculoskeletal system and connective tissue were less popular causes (Table 2).

### Demographic characteristics of the deceased mothers in Iran

A summary of the demographic characteristics of the mothers who died due to maternity reasons, included in this review study, are described in the second column of Table 3. Out of the 29 studies, 6 studies did not report any demographic information for died mothers, and of the remaining, only 12 studies examined the association between demographic characteristics and death.

**Table 3:** Frequency (%) of characteristics of died mothers and the results of analyses on factors ASSOCITED or NOT ASSOCIATED with maternal death in Iran

Characteristics of dead mothers	N (%)^a^	Studies found associations		Studies found NO association	
		N of cases^b^	Ref	N of cases^b^	Ref
Maternal age	*N* = 1990 (42.9%) Mean = 30.02 y	303	[40, 45, 56]	363	[24, 42, 46, 48, 57]
Gravidity	*N* = 735 (15.9%) Mean = 3.67	486	[24, 42, 45, 54, 56, 57]	66	[55]
Parity	N = 70 (1.5%) Mean = 1.7	–	–	–	–
Type of delivery • Normal • Caesarean section • Others (nor clear)	*N* = 4157 (89.8%) 35% 55% 10%	2408	[38, 42, 48, 50, 55, 57]	241	[24, 40, 45]
Period of death • During pregnancy • Childbirth • Postpartum • Unclassified & Unknown	*N* = 2310 (49.9%) 13% 4% 70% 13%	165	[46, 55]	106	[40, 48]
Region of residence • Rural/Nomadic • Urban	*N* = 1431 (30.9%) 55% 45%	185	[36, 46, 55]	538	[24, 40–42, 48, 50, 51]
Education • Illiterate/Primary • Literate	*N* = 3708 (80.0%) 32% 68%	1992	[38, 51, 54–56]	55	[57]
Location of birth • Hospital/ Labor facilities • In the way/ Home	*N* = 2153 (46.5%) 88% 12%	1889	[38, 48]	89	[24, 57]
Location of death • Hospital/ Labor facilities • In the way/ Home • Unknown	*N* = 1858 (40.1%) 72% 14% 14%	373	[50, 55]	197	[40, 42, 48]
Birth attendant • Skilled • Trained midwife • Untrained/local midwife • Other (Unknown/Undelivered)	*N* = 2632 (56.8%) 83% 3% 9% 6%	2167	[38, 48, 50]	219	[24, 45]
Pregnancy intentions • Wanted • Unwanted • Unknown & Intermediate	*N* = 532 (11.5%) 64% 27% 9%	–	–	–	–
Economic status • High • Middle • Low	*N* = 993 (21.4%) 5% 5% 90%	–	[41]	–	–
Occupation • Employed • Unemployed	*N* = 1189 (25.7%) 14.2% 85.8%	–	[51]	–	–
Prenatal care • Yes • No	*N* = 2083 (45.0%) 91.5% 8.5%	1860	[38, 57]	99	[46]
Type of insurance • Public social security • Public health service • Private	N = 109 (2.4%) 50% 34% 16%	–	–	–	–
Supplemental insurance • Yes • No	N = 109 (2.4%) 27% 73%	–	–	–	–

^a^N indicates number of cases (death) in each of the determined characteristics in all the included studies and % was calculated by dividing N to 4633, which is the total number of reported maternal deaths in Iran based on this systematic review

^b^These numbers indicate the total number of deaths in the included studies with the information on their association with the determined characteristics

As shown in the Table 3, the mean of age, gravity and parity of the mothers included in the studies were 30 years, 3.67 and 1.7 respectively. Out of the total reviewed maternal deaths in this study (*n* = 4633), the type of delivery in 4157 cases (90%) was reported, which was 55% C-section, 35% vaginal delivery and others not reported. Similarly, out of 1431 deaths, 55% occurred in rural or nomadic areas rather than urban areas and 70% of 2310 cases happened at postpartum period, as against 17% which happened during pregnancy or childbirth. Additionally, 32% of mothers were illiterates, 86% were unemployed and 90% had poor economic status when compared to the middle or high-class groups. In 88 and 72% of deaths respectively, the birth location and the death location were hospital or labor facilities rather than home or other places. Moreover, 83% of the mothers had skilled birth attendants and 91% utilized prenatal care services. The information on pregnancy intention was available in 12% of all the mortalities, indicating that 36% of the pregnancies were unwanted. However, more detailed information on and the characteristics less reported of the dead mothers are shown in Table 3.

### Determinants of maternal mortality in Iran

The associations between several factors (*n* = 12) and the maternal mortality were examined in almost 55% of the studies included in this review, a summary of the results, and their references are shown in Table 3. The association of ‘maternal age’ with mortality was examined in 8 studies, of which 5 studies did not find a significant effect (363 vs. 303 deaths). In contrast, ‘gravidity’, which was examined in 7 studies was discovered to be a significant factor associated with mortality in 6 studies (*n* = 486). The ‘type of delivery’, was another factor which was examined frequently in the Iranian studies (*n* = 9 studies) and were strongly in favor of an association between caesarean section and mortality (2408 cases associated vs. 241 not associated). Contrarily, ‘region of residence’, which also was examined frequently (*n* = 10 studies), did not show an association with mortality in most of the cases (538 cases not associated vs. 185 associated).

The association between ‘socioeconomic status’ of the mothers with their mortality was also examined in several studies; and a strong association was found between ‘education’ and mortality in 5 studies with 1992 aggregated cases versus 1 study with 55 cases indicating no association. ‘Economic status’ and ‘occupation’ of the mothers were also both d to be associated with mortality in 2 studies. Additionally, ‘locations of birth and death’, specifically, locations of birth were also revealed to be associated with mortality in 1889 samples and in 373 versus 89 and 197 cases respectively. ‘Skilled birth attendants’ versus unskilled attendants were also significant factors in association with death in 2167 versus 219 cases indicating no association. Similarly, having ‘prenatal care’ in 2 studies with aggregated 1860 samples versus 1 study with 99 cases was also an effective factor in mortality of the mothers. Finally, ‘period of death’ (during pregnancy, childbirth, postpartum and others), which were examined in 4 studies provided inconsistent results.

Other factors discovered to be associated or not associated with maternal mortality in this review study, but studied less frequently in Iran were as follows were summarized in Table 4.

**Table 4:** Studies with evidence on less studied factors Associated or NOT Associated with maternal mortality in Iran

Determinants	associated	Not associated
Preterm of gestational age	[45]	–
Obesity	[45]	–
Quality of care	[54]	–
Immune pregnancy ratio	[54, 56]	–
Human development index	[53]	–
Gender inequality in education	[53]	–
Access to improved water source	[38]	–
Fertility rate	[41]	–
Number of midwives	–	[51]
Gini coefficient	–	[22, 53]
Attendance of girls in secondary schools	–	[6, 38]
Numbers of hospital beds	–	[41]
Number of physician	–	[41]
Use of improved sanitation	–	[38]

## Discussion

This review study, as mentioned earlier, is the first one that systematically reviewed both the determinants of maternal mortality and the causes using the WHO framework of ICD-MM in the context of Iran. The results reported here is based on 29 original studies with various designs which have studied either causes or determinates of maternal mortality or both in cities or provinces of Iran or at the country level in a specific groups of mothers or in general population (Table 1). Therefore, due to the diversity of the studies in terms of their main objective, study population, design, geographical areas, and publication dates, the formal meta-analysis to estimate the effect size of the determinants on maternal death was not possible. Additionally, methodological limitations of the original studies included in this review, including the dominance of cross-sectional studies which generally indicate associations between variables rather than causality, and poor quality scores of some of the included studies, reduce the level of confidence on the generalizability of these results to the maternal mortality cases in the whole country. However, in the lack of an updated original national level study, the result of a systematic review could be of the highest value and applicability for the policy makers. Understanding the context-based causes and determinants of maternal deaths is critically important for development of specific interventions for each groups of mothers or areas in accordance with their priorities to more effectively reduce the burden of mortality and morbidity in each area.

In terms of the causes of maternal deaths in Iran, the result of this systematic review indicated that of the total 2655 deaths reviewed, the causes of 69.9 and 20.6% of cases respectively were direct and indirect, and the causes of 5.2% of cases were unspecified, in addition to 4.3% of cases, with unclear cause (Table 2). The results of the causes of maternal death in this systematic review is almost similar to the study of Dadipoor et al. [13], the only review study conducted in Iran before this review, due to sharing some of the original studies in these two reviews. Dadipoor et al., however, has classified all the causes only to direct and indirect, not mentioning the basis of their classification, and reported that 74% of the mortalities in Iran had direct causes and remaining were due to indirect causes. Also, categories that they used as direct causes and also as indirect causes were less comparable with the present review. Comparing the frequency of causes of death resulted from our review with average of the world or other regions, based on the study of Say et al. [29], which is the only comprehensive global study using ICD-10, (although classification is in two categories as direct and indirect rather than four categories) indicates that direct causes in our study are slightly lower than the average of the world (70% vs. 72%) (Table 5).

**Table 5:** Percentage of the causes of maternal mortality in this review and comparing with regions of the world

Causes Regions	Direct causes						Indirect causes	Other^a^	Total	Total number of maternal deaths
	Abortion	Embolism	Hemorrhage	Hypertension	Sepsis	Other direct causes				
Iran (based on this review)	2%	5%	31%	17%	8%	6%^b^	21%	10%	100%	2655
Worldwide	8%	3%	27%	14%	11%	10%	28%	–	100%	2,443,000
Developed Regions	8%	14%	16%	13%	5%	20%	25%	–	100%	14,590
Developing Regions	8%	3%	27%	14%	11%	10%	28%	–	100%	2,428,000
Northern Africa	2%	3%	37%	17%	6%	17%	18%	–	100%	22,410
Sub-Saharan Africa	10%	2%	25%	16%	10%	9%	29%	–	100%	1,310,000
Eastern Asia	1%	12%	36%	10%	3%	14%	25%	–	100%	56,320
Southern Asia	6%	2%	30%	10%	14%	8%	29%	–	100%	783,000
Southeastern Asia	7%	12%	30%	14%	6%	14%	17%	–	100%	147,100
Western Asia	3%	9%	31%	14%	5%	16%	23%	–	100%	28,860
Caucasus and central Asia	5%	11%	22%	15%	9%	17%	22%	–	100%	5400
Latin America and Caribbean	10%	3%	23%	22%	8%	14%	19%	–	100%	69,000
Oceania	7%	15%	29%	14%	5%	13%	17%	–	100%	4080

Ref for global data and data of the regions of the world is: Say, L. et al., Global causes of maternal death: a WHO systematic analysis. The Lancet Global Health, 2014. 2(6): p. e323-e333 [29]

^a^Other category included unknown, and unclassified. ^b^Six percent of other direct causes is the sum of unanticipated complications of management and other obstetric complications excluded embolism in order to compare with the MDG regions cause of death at this table

31% of all causes in this review and 35% in the study of Dadipoor et al. [13] showed that hemorrhage is the leading cause of maternal death in Iran [13]. Although, it is the most common cause of maternal deaths everywhere, its proportion among all the causes is higher in Iran than the rest of the world (27%) with 16% of the developed regions which is lower than Eastern Asia and Northern Africa (Table 5). Nevertheless, no in-depth study has investigated the reasons why, despite various interventions conducted in Iran, hemorrhage is still the leading cause of death. However, there is a study in Iran [30] stating that three main factors that lead to hemorrhage and mortality in Iran are: doctor’s delayed decision-making, delayed transfer to primary care center and delayed emergency care provision; although the last cause was revealed to be more important than the other two. According to Say et al. [29], in the world level, these reasons are also unknown and with the available data, it is not possible to establish the justifications for the persistence of hemorrhage as the leading cause of death. They, however, assumed that, it may be as a result of increasing rates of caesarean section, which is also very high in Iran comprising half of all the deliveries. Therefore, they argue that abortion and obstructed labor may be misclassified and will erroneously increase the hemorrhage category.

Deaths attributed to hypertensive disorders, are the second highest both in Iran (17%) and worldwide (14%) among all direct causes and the most prominent cause in the Latin America and Caribbean region (22%). However, death due to the hypertension in Iran is highest among all world’s regions except Latin America and Caribbean region, therefore, this needs special attention [29]. It seems that having adequate and timely prenatal care could highly be effective in early detection of hypertension and control its consequences, particularly in developing world [13]. Abortion, another direct cause of death, however, has been reported to be less prevalent in Iran compared to the worldwide (2% vs. 8%) and almost all the regions shown in Table 4. While this might be attributed to generally having low rate of abortion in Iran due to the religious and legal restrictions, for the same reason, these deaths could also have been reported as hemorrhage or infection without documenting the underlying cause.

Indirect causes, as shown in Table 4, are, however, comparably less prevalent in Iran than the rest of the world (21% vs. 28%) but slightly higher than North Africa and Southeastern Asia. Nevertheless, this to certain regards might be due to the classification of causes to four groups in our review compared with only two groups in the study of Say et al. [29]. Of the indirect causes of death in Iran, ‘circulatory system diseases’ was the most prevalent cause (8% of maternal mortalities), which needs special attention in research and practice. As direct causes decrease as a result of specific interventions, indirect causes should also be refocused to reduce maternal mortality [29].

Furthermore, there is evidence that the major causes of maternal mortality in Iran, as argued by Moazzeni [3], is experiencing a shift from those more commonly seen in developing nations such as infection and hemorrhage to those that are more common in developed countries such as pulmonary embolism and stroke. This issue is important for considerations in policy making and follow ups should be taken to check the shift of the causes of death. Understanding the context-based causes of maternal deaths is critically important for development of specific interventions to reduce the burden of mortality and morbidity in each area, in accordance with the causes with high priority. Similarly, effectiveness of the previous and current policies and strategies to overcome these causes should be evaluated, and according to the results, further policies with higher effectiveness should be developed.

With regard to the distribution of characteristics among the deceased mothers, in comparison to lived mothers in Iran, although the comparison is very difficult due to the variations in study dates and study populations, it seems that the deceased mothers in comparison to other mothers were more likely to have C-section, dye in postpartum period, less utilization of maternity care, more likely to be poor, from rural areas, illiterate, less educated, unemployed (Table 3). However, the aggregated results of analyses included in this review (55% of all the included studies), provided evidence that gravidity, type of delivery, socioeconomic status of mothers, location of birth and death, having skilled birth attendant and prenatal care are associated with the maternal death, while the present review failed to find evidence for the association of maternal age and region of residence (urban/rural) with maternal death. The results of determinants of maternal death in Iran were mainly compatible with most of the available evidence in the world. For example, having C-section delivery in most of the studies are associated with higher death among mothers mostly due to being associated with more bleeding and hysterectomy in mothers with C-section delivery [31–33]. The results of the present review in terms of socioeconomic status of mothers, location of birth and death, having skilled birth attendant and prenatal care were also in line with most of the global evidence [31–33]. However, despite the finding of this review, most literature indicated the higher rate of death among rural mothers compared to mothers living in urban areas [31–34]. This might be because of high availability of primary health care for Iranian mothers living in rural areas as living in urban areas, in contrast the global situation in average.

We believe this review has several strengths; this is the first study that comprehensively reviewed all the available published and unpublished sources on both cause and determinants of maternal death in Iran using the ICD-MM methodology, its advantages discussed earlier. Our search strategy was extensive and reproducible, as required by systematic reviews of published work. We identified many national reports and special surveys that might not have been accessible otherwise. We did a transparent methodological quality assessment and attempted to keep the risk of bias due to methodological limitations to a minimum by removing the poor quality studies. However, as other studies, this review is also subject to several limitations. In addition to those that are explained at the beginning of this section, several studies included here were not clear enough on the cause of maternal death, forcing us to add another category, as unclassified, to the original ones defined in ICD-MM. Similarly, the report of the results in this review was based on what the original studies also reported, the quality of which depends on how they completed the death certificate, how they interpreted the information from the death certificate, and whether medical records were available for review to check the determined cause of death.

## Conclusions

Understanding the causes of and factors contributing to maternal deaths is critically important for development of interventions and funding to reduce maternal deaths. The results of this review study highlighted the importance of attention toward the causes of maternal deaths in Iran with higher proportions. Specifically, maternal death due to hemorrhage and then hypertension needs special attention, as these two causes are responsible for half of the all deaths of Iranian mothers.

This study also summarized the results of analyses examined the association between maternal mortality and characteristics of the mothers in Iran, and indicated that gravidity, type of delivery, period of death, socioeconomic status of mothers, place of birth and death, and utilization of maternity care are associated with death. In addition to a need for a national study to determine factors associated with mother’s death, specific differences in the determinants of death among the areas and provinces of the county should be considered.

Another concern is the diversity of classification of causes of maternal death in Iran and lack of a universal way in diagnosis and classification, as identified as a limitation of the primary studies included in this review. Further efforts are needed to improve the classification of deaths and availability and quality of data related to maternal mortality. We applied ICD-MM methodology as a preferred one by the WHO for classification of maternal deaths during pregnancy, childbirth and the puerperium reviewed in this study. To avoid misclassification of causes of death and many advantages of using ICD-MM, it is highly recommended that this methodology is applied as a guide in whole country, although in recent years in has reached to the attention of policymakers.

## Abbreviations

CS: Cesarean section
ICD-MM: International classification of diseases- maternal mortality
MDGs: Millennium development goals
MMR: Maternal mortality ratio
ND: Normal vaginal delivery
WHO: World health organization
XS: Cross sectional
